# Toll-like receptor 9 suppresses lupus disease in *Fas*-sufficient MRL Mice

**DOI:** 10.1371/journal.pone.0173471

**Published:** 2017-03-09

**Authors:** Kevin M. Nickerson, Yujuan Wang, Sheldon Bastacky, Mark J. Shlomchik

**Affiliations:** 1 Department of Immunology, University of Pittsburgh, Pittsburgh, Pennsylvania, United States of America; 2 Department of Pathology, University of Pittsburgh, Pittsburgh, Pennsylvania, United States of America; Instituto Nacional de Ciencias Medicas y Nutricion Salvador Zubiran, MEXICO

## Abstract

Genetic deficiency in TLR9 accelerates pathogenesis in the spontaneous polygenic MRL.*Fas*^*lpr*^ murine model of systemic lupus erythematosus, despite the absence of anti-nucleosome autoantibodies. However, it could be argued that this result was dependent on *Fas*-deficiency rather than lupus-promoting genes in the MRL genetic background. Here we report the effects of TLR9 deficiency on autoimmune disease independent of the *lpr* mutation in *Fas* by characterizing *Tlr9*^*-/-*^ and *Tlr9*^*+/+*^ mice on the *Fas*-intact MRL/+ genetic background. By 30 weeks of age, *Tlr9-*deficient MRL/+ had more severe renal disease, increased T cell activation, and higher titers of anti-Sm and anti-RNA autoantibodies than *Tlr9*-intact animals, as had been the case in the MRL.*Fas*^*lpr*^ model. In addition, *Tlr9*-deficient MRL/+ mice had increased numbers of germinal center phenotype B cells and an increase in splenic neutrophils and conventional dendritic cell populations. Thus, the disease accelerating effects of *Tlr9* deficiency are separable from those mediated by the *Fas* mutation in the lupus-prone MRL genetic background. Nonetheless, disease acceleration in *Tlr9*-deficient MRL/+ mice was phenotypically distinct from that in *Fas*-deficient counterparts, which has important implications.

## Introduction

Systemic lupus erythematosus (SLE) is an autoimmune disorder characterized by the production of autoantibodies, especially against nucleic acid associated self-antigens. The current paradigm for why nucleic acid containing self-antigens are highly prone to break self-tolerance is that these antigens are able to directly activate autoantigen-specific B cells via co-ligation of the autoreactive B cell receptor together with a nucleic acid responsive Toll-like receptor, TLR7 or TLR9 [1, 2]. Although autoreactive B cell receptors are generated at a high frequency as a consequence of V(D)J recombination, and B cells do express TLR7 and TLR9, most individuals do not make significant titers of autoantibodies or progress to end-organ disease due to self-tolerance mechanisms including those that delete, edit or functionally inhibit autoreactive clones prior to entry into the mature B cell repertoire [3–5].

SLE in most patients is driven by the additive or synergistic effects of multiple lupus susceptibility alleles that individually confer low disease risk [6]. Similarly, animal models of SLE can be categorized as spontaneous polygenic models (for example, MRL.*Fas*^*lpr*^, NZBxW, or the NZM series) driven by multiple alleles, spontaneous monoallelic models (such as B6.*yaa*) driven by single high penetrance alleles on an otherwise non-autoimmune genetic background, or inducible models such as the SLE-like disease produced by *i*.*p*. provision of pristane in mice that are otherwise non-autoimmune [7].

Previously we showed that *Tlr9*, the endosomal sensor for DNA, is necessary for anti-nucleosome autoantibody production in the spontaneous polygenic MRL.*Fas*^*lpr*^ (or MRL/lpr) mouse model of SLE [8–10]. Similarly, *Tlr7*, the endosomal sensor for RNA, was necessary for production of anti-Sm and anti-RNA autoantibodies [9, 10]. Mice deficient in both *Tlr7* and *Tlr9*, or deficient in *Myd88*, did not produce autoantibodies of either specificity [10]. MRL.*Fas*^*lpr*^ deficient in *Myd88* or *Tlr7* had reduced clinical disease, while those deficient in *Tlr9* unexpectedly had significantly exacerbated disease, suggesting that *Tlr9* suppresses development of clinical pathology in lupus [8–10] despite its paradoxical role in breaking tolerance in anti-nucleosome and anti-DNA B cells. The exacerbation of disease in *Tlr9*^*-/-*^ MRL.*Fas*^*lpr*^ was dependent on both *Tlr7* and *Ifnar1*, suggesting that intact *Tlr9* inhibits a proinflammatory signaling axis on the lupus-prone genetic background [10, 11]. Deletion of *Myd88* specifically in B cells or dendritic cells subsequently demonstrated distinct roles for innate immune signaling in different cell lineages [12]. Genetic deletion of non-endosomal *Tlr2* and *Tlr4* resulted in a reduction of disease in the monoallelic B6.MRL-*Fas*^*lpr*^ (B6/*lpr*) model but did not affect disease on the MRL.*Fas*^*lpr*^ genetic background [13, 14]. Targeting the TLR pathways is an area of active investigation in human SLE and other rheumatic diseases [15].

Acceleration of disease in the absence of *Tlr9* has been demonstrated in several other spontaneous models of SLE, all of which so far reported were monoallelic models derived from the C57BL/6 background. B6.MRL-*Fas*^*lpr*^ mice lacking *Tlr9* had more severe splenomegaly, proteinuria and glomerulonephritis and displayed a shift in autoantibody profiles from homogenous to nucleolar HEp-2 antinuclear antibody (ANA) staining [16]. Similarly, B6.*Nba2* and B6.*Nba2*.*yaa* mice had decreased anti-nucleosome IgG titers but more severe renal disease when *Tlr9* was absent [17]. B6.*FcgrIIb*^*-/-*^ mice lacking *Tlr9* had reduced anti-nucleosome autoantibodies and accelerated mortality [18]. B6.*Plcg2*^*Ali5/Ali5*^ bearing a gain of function mutation in phospholipase c gamma 2 had decreased anti-nucleosome autoantibodies and more frequent nucleolar ANA patterns with more severe glomerulonephritis when *Tlr9* was absent [19]. Discordant regulation of RNA and DNA autoantibodies and disease by B-cell specific *Tlr7* and *Tlr9* was also recently demonstrated in a B6.*Was*^*-/-*^ B cell chimeric model in which the Wiscott-Aldrich protein deficiency promotes lupus-like disease [20]. *Tlr9* deficiency also accelerated pristane-induced disease on the BALB/c background [21] but either prevented induction of lupus disease [22] or had no effect [23] when pristane was administered to relatively resistant C57BL/6 mice, likely reflecting both the importance of genetic background as well as different routes to inflammation in an inducible rather than a spontaneous disease model.

Despite these several examples in which *Tlr9* deficiency exacerbated lupus disease in monoallelic models, we are unaware of any other examples of *Tlr9* deficiency in a spontaneous polygenic lupus model. The MRL.*Fas*^*lpr*^ model meets all of the American College of Rheumatology (ACR) criteria for a clinical SLE diagnosis, including skin and renal involvement and production of autoantibodies against both DNA and RNA components—including a high frequency of autoantibodies against the Sm antigen, which are not observed or rarely observed in many monogenic and even some polygenic lupus models [24]. Nonetheless, the MRL.*Fas*^*lpr*^ model has often been criticized due to the presence of the *lpr* mutation in *Fas*, which significantly accelerates disease, resulting in appearance of autoantibodies as early as 8 weeks of age with 50% mortality by 4–5 months [25–27].

Fas signaling is complex, promoting outcomes that may include not only apoptosis but also in some cases proliferation or inflammatory cytokine production, via pathways that could conceivably interact directly or indirectly with TLR signaling [28, 29]. Further, Fas is expressed on multiple cell types, many of which have been directly implicated in immune activation when Fas is deleted or inactive [30]. For example, we recently demonstrated that Fas and FasL expression on T cells, promoted by cDCs, regulates the autoreactive extrafollicular B cell response to TLR9-containing IC ligands by culling extrafollicular helper T cells; this mechanism of autoreactive B cell regulation would be unavailable in Fas-deficient animals such as the MRL.*Fas*^*lpr*^ strain [31]. Although in some cases others and we have found that key genetic deletions in autoimmune models that were accelerated by Fas deficiency had overall similar phenotypes in Fas-sufficient congenic animals [32–34], this has mainly not been investigated. Particularly considering that TLR signaling and Fas could interact, either directly or indirectly, we could not exclude the real possibility that some of the disease-modulating effects of *Tlr9* deficiency were due to genetic interactions with and immune dysregulation mediated by the *lpr* mutation of *Fas*. Therefore, we generated *Tlr9*^*-/-*^ mice on the congenic MRL/+ background. MRL/+ mice have functional *Fas* but do develop autoantibodies and clinical pathology, albeit at a later age than MRL.*Fas*^*lpr*^. Here we show that *Tlr9*^*-/-*^ MRL/+ have significantly accelerated clinical pathology and a shift toward RNA-reactive autoantibody profiles without anti-nucleosome autoantibodies, recapitulating all of the major phenotypes observed in the MRL.*Fas*^*lpr*^ model. In addition, we observed an increase in the frequency of germinal center B cells and an expansion of splenic neutrophils and conventional dendritic cells. Thus, *Tlr9* regulates lupus autoantibody production and disease in a spontaneous polygenic lupus model independent of the disease-accelerating effects of the *Fas*^*lpr*^ mutation.

## Materials and methods

### Mice

Previously described *Tlr9*^*-/-*^ MRL.*Fas*^*lpr*^ mice [10] were crossed for one generation to *Fas*-sufficient MRL/MpJ (Jackson labs stock #000486). These F1 progeny were intercrossed to generate experimental cohorts that included both *Fas*^*lpr/+*^ and *Fas*^*+/+*^ mice that included all possible *Tlr9* genotypes; *Fas*^*+/+*^ progeny of this generation were further intercrossed to generate additional experimental cohorts of *Fas*^*+/+*^ animals. Except where noted, no evidence of a *Fas* haploinsufficient phenotype was observed and thus data are pooled from both cohorts. Experimental cohorts were analyzed at 30 weeks of age. Since the kinetics of disease progression differs with gender in the MRL/+ and MRL.*Fas*^*lpr*^ lupus models, only female mice were included in the analysis.

This study was carried out in accordance with the recommendations in the Guide for the Care and Use of Laboratory Animals of the National Institutes of Health and the policies and procedures of the University of Pittsburgh Division of Laboratory Animal Resources. The protocol was approved by the Institutional Animal Care and Use Committee of the University of Pittsburgh under protocol number 13102426. All efforts were made to minimize suffering. Mice were housed under specific pathogen free conditions. Animals were monitored daily by an experienced observer. Early humane endpoints as predetermined in the experimental protocol included any of the following criteria: animals which a) are hunched and do not move on gentle prodding; b) have severe skin lesions (autoimmune dermatitis) covering more than approximately 1/2 of the back or 1/4 of the body; c) are otherwise judged to be pre-moribund by the observer. No animals in this experimental cohort met these criteria or died of any cause prior to the experimental endpoint. Anesthesia or analgesia were not required. Animals were euthanized by CO2 inhalation followed by cervical dislocation and/or removal of a vital organ(s).

### Evaluation of clinical disease and autoantibodies

Proteinuria, glomerulonephritis, interstitial renal disease, and dermatitis were evaluated exactly as previously described [11] by individuals blinded to the genotypes of the animals.

HEp-2 antinuclear antibody assays were performed using Kallestad HEp-2 slides (Bio-Rad, Hercules, CA) as a substrate. Serum samples were diluted to 1/100 in 1x PBS, 1% BSA, 0.05% sodium azide, and bound autoantibodies were detected using goat anti-mouse IgG-FITC (Southern Biotech, Birmingham, AL). Samples were scored for the dominant staining pattern, presence or absence of mitotic chromatin staining, and relative cytoplasmic staining intensity by an individual blinded to the genotypes of the samples.

Autoantibody and total IgM and IgG ELISAs were performed essentially as previously described [11]. Samples were assayed over a total of eight three-fold dilutions starting at 1/100 for anti-nucleosome and anti-Sm ELISAs, four three-fold dilutions starting at 1/100 for anti-RNA and anti-IgG2a rheumatoid factor ELISAs, and eight three-fold dilutions starting at 1/5000 for total IgM ELISA or 1/50000 for total IgG ELISA. ELISA standards were purified PL2-3 and BWR4 antibodies, hybridoma supernatants from Y2 and 400tμ23 cultures, or purified unconjugated IgM,κ and IgG1,κ isotype control antibodies (BD Biosciences, Franklin Lakes, NJ).

### Flow cytometry

Single cell splenocyte suspensions were prepared and stained in ice-cold PBS with Ghost Dye Violet 510 (Tonbo Biosciences, San Diego, CA) to exclude dead cells. Surface staining was subsequently performed in PBS containing 3% FCS and 5 mM EDTA in the presence of unconjugated anti-CD16/32 clone 2.4G2 (purified in-house). Antibody clones used for staining were directed against CD45R (RA3-6B2), CD23 (B3B4), CD19 (1D3), CD11c (HL3) and TCRß (H57-597) (BD Biosciences, Franklin Lakes, NJ); CD62L (Mel-14), CD138 (281–2), CD11b (M1/70), TCRß (H57-597) and I-A/I-E (M5/114.15.2) (BioLegend, San Diego, CA); SiglecH (eBio440c) (eBioScience, San Diego, CA); and Ly6G (1A8), CD45R (RA3-6B2), CD19 (1D3), CD21/35 (7G6), CD317 (927), CD44 (Pgp1), CD8 (TIB105), and CD4 (GK1.5) (purified and fluorophore-conjugated in-house). Peanut agglutinin (PNA) was from Vector Laboratories (Burlingame, CA). All antibodies and PNA were titered prior to experimental use. Cells were fixed with 1xPBS 1% paraformaldehyde. Flow cytometry data was collected on a BD LSRII or BD Fortessa and analyzed in FlowJo (Tree Star, Ashland, OR).

### Microscopy

Seven-micrometer sections of OCT-embedded frozen spleens were prepared and stained as previously described [35]. Sections were stained with fluorophore-conjugated anti-Bcl6 (clone K112-91, BD Bioscience, Franklin Hills, NJ); anti-IgD (clone 11-26c, eBioscience, San Diego, CA); and/or PNA, anti-CD4 (clone GK1.5), anti-CD19 (clone 1D3.2), anti-kappa (clone 187.1) and/or anti-F4/80 (clone F4/80) (purified and conjugated in-house).

Immunofluorescent images were acquired on an Olympus IX83 microscope with a 20x Plan Apo 0.75 NA objective and Hamamatsu ORCA-Flash4.0 CMOS monochrome camera in Cellsens Dimension software. Spleen images were obtained as tiled multifield images and were cropped and converted to 8-bit RGB in Cellsens software for display.

### Statistical analysis

Horizontal lines on dot plots indicate the median value. Statistical comparisons were made by pairwise two-tailed Mann-Whitney U-test or Fisher's exact test. Significance values were calculated by GraphPad Prism software.

## Results

**Fig 1:**
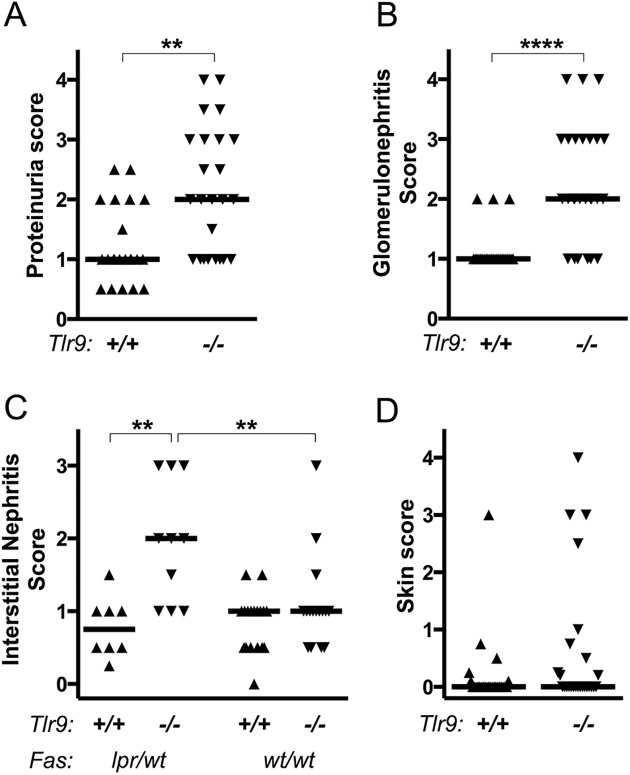
Renal disease is suppressed by *Tlr9* in MRL/+ mice. **(A)** Proteinuria was evaluated in 30 week old mice of the indicated genotypes by dipstick assay. **(B)** Severity of glomerular disease on H&E stained sections was evaluated by a pathologist on a 0–6 scale. **(C)** Perivascular and interstitial renal infiltrates were evaluated by a pathologist on a 0–4 scale. **(D)** Skin lesions were scored based on area with up to an additional 0.5 points for facial rash / loss of whiskers and 0.25 points for dermatitis of each ear. In all graphs, horizontal lines represent medians and each point represents an individual animal. ** p<0.01; **** p<0.0001 by two-tailed Mann-Whitney U-test. Data are pooled from five experimental cohorts.

We first characterized clinical features of SLE. *Tlr9*-deficient MRL/+ mice had significantly more proteinuria than *Tlr9*-intact MRL/+ (Fig 1A). *Tlr9*^*-/-*^ MRL/+ mice had notable glomerulonephritis at 30 weeks of age, a time point at which the majority of *Tlr9*^*+/+*^ MRL/+ mice had not yet developed any significant renal disease (Fig 1B). Small areas of lymphocytic infiltrates, restricted for the most part to perivascular regions, were observed in most experimental animals of either genotype by 30 weeks of age, but significant tubular interstitial infiltrates were observed only in the *Tlr9*^*-/-*^ mice, especially those in which *Fas* was heterozygous for the *lpr* mutation (Fig 1C). Thus, the absence of *Tlr9* significantly accelerates the onset of glomerular renal disease and interstitial nephritis in *Fas*-sufficient MRL lupus-prone mice. In contrast to the renal phenotype, by 30 weeks of age few MRL/+ animals of either *Tlr9* genotype had developed significant dermatitis (Fig 1D; 1 of 26 *Tlr9*^*+/+*^ and 5 of 26 *Tlr9*^*-/-*^ animals with skin score > = 1).

**Fig 2:**
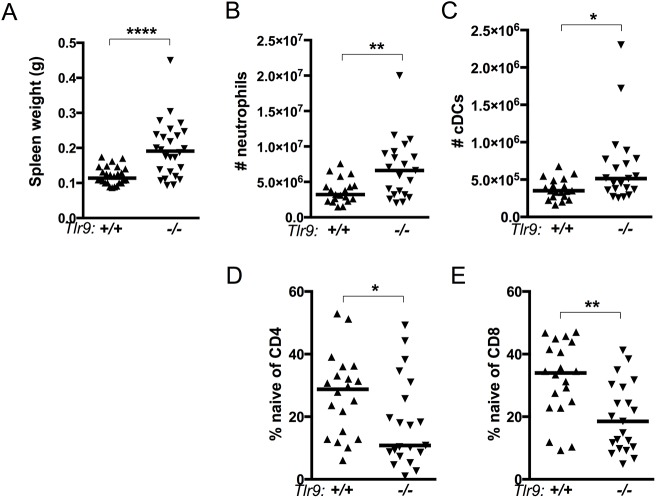
Increased spleen weight and T cell activation in *Tlr9*^*-/-*^ MRL/+ mice. **(A)** Spleens were weighed. **(B)** Ly6G^+^CD11b^+^ neutrophils and **(C)** CD19^-^CD11c^+^I-A/I-E^+^ dendritic cells were evaluated by FACS. **(D-E)** Naive (CD44^-^CD62L^+^) cells were evaluated by FACS among TCRß^+^CD4^+^ (B) and TCRß^+^CD8^+^ (C) populations. * p<0.05; ** p<0.01; **** p<0.0001 by two-tailed Mann-Whitney U-test. Data are pooled from five (A) or four (B-E) experimental cohorts.

Previously we observed that *Tlr9*^*-/-*^ MRL.*Fas*^*lpr*^ mice had significantly worse splenomegaly compared to *Tlr9*^*+/+*^ MRL.*Fas*^*lpr*^ [8–10]. Splenomegaly in the MRL.*Fas*^*lpr*^ strain is dominated by the expansion of a population of CD3^+^B220^+^CD4^-^CD8^-^ T cells (DN T cells) which may represent a population of activated T cells that escaped *Fas*/*FasL*-mediated activation induced cell death [36, 37] and that are not observed in the MRL/+ strain. Indeed, we did not observe significant numbers of DN T cells in *Tlr9*^*-/-*^ MRL/+ spleens (not shown). Surprisingly, *Tlr9*^*-/-*^ MRL/+ spleens were nonetheless larger than those in *Tlr9*^*+/+*^ animals (Fig 2A), independent of the *Fas*^*lpr*^ mutation. While the total number of T cells, B cells and plasmacytoid dendritic cells were unchanged with *Tlr9* genotype, there was a modest expansion in total CD11c^+^ MHCII^+^ conventional dendritic cells and a roughly two-fold increase in Ly6G^+^CD11b^+^ neutrophil numbers in *Tlr9*^-/-^ MRL/+ mice (Fig 2B and 2C, S1 Fig).

There were also substantial differences in the activation status of both CD4^+^ and CD8^+^ compartments. *Tlr9*^*-/-*^ MRL/+ had a significant decrease in the proportion of T cells with a CD44^low^CD62L^high^ naive phenotype (Fig 2D and 2E), and a concomitant increase in the proportion of CD4 and CD8 cells with a CD44^high^CD62L^low^ effector memory phenotype (not shown). This shift in activation phenotypes was similar to that observed in MRL.*Fas*^*lpr*^ mice [10].

**Fig 3:**
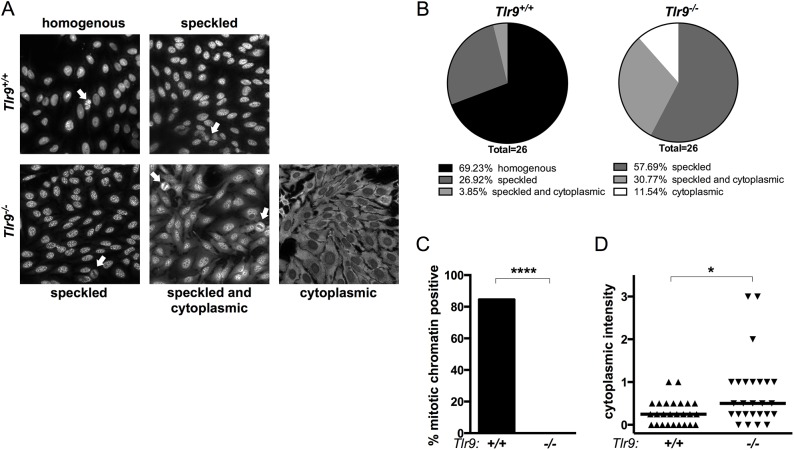
HEp-2 antinuclear antibody staining patterns are changed in *Tlr9*^*-/-*^ MRL/+ mice. **(A)** Representative HEp-2 staining patterns from *Tlr9*^*+/+*^ MRL/+ (*top*) and *Tlr9*^*-/-*^ MRL/+ (*bottom*) serum. **(B)** Proportion of sera with the indicated dominant staining pattern from *Tlr9*^*+/+*^ MRL/+ (*left*) or *Tlr9*^*-/-*^ MRL/+ (*right*). **(C)** Percentage of sera of indicated genotypes which stained positive for mitotic bodies in the HEp-2 ANA assay. **** p<0.0001 by two-tailed Fisher's exact test. **(D)** Relative intensity of cytoplasmic staining in the HEp-2 ANA assay. * p<0.05 by two-tailed Mann-Whitney U-test.

We tested the serum of *Tlr9*^*+/+*^ and *Tlr9*^*-/-*^ MRL/+ for autoantibodies by first screening on HEp-2 cells. *Tlr9*^*+/+*^ MRL/+ mice displayed homogenous or speckled nuclear staining patterns consistent with the presence of anti-DNA autoantibodies (Fig 3A and 3B). In contrast, no *Tlr9*^*-/-*^ MRL/+ animals had homogenous nuclear HEp-2 staining. Instead, *Tlr9*^*-/-*^ MRL/+ serum produced only speckled (nucleolar), speckled with cytoplasmic, or cytoplasmic-only staining patterns, suggestive of anti-RNA specificities. Most *Tlr9*^*+/+*^ sera but no *Tlr9*^*-/-*^ sera stained mitotic Figs in the HEp-2 ANA (Fig 3C). As was the case in MRL.*Fas*^*lpr*^ [10], both the frequency and relative intensity of cytoplasmic staining HEp-2 reactivities were increased when *Tlr9* was absent in the MRL/+ mice (Fig 3D).

**Fig 4:**
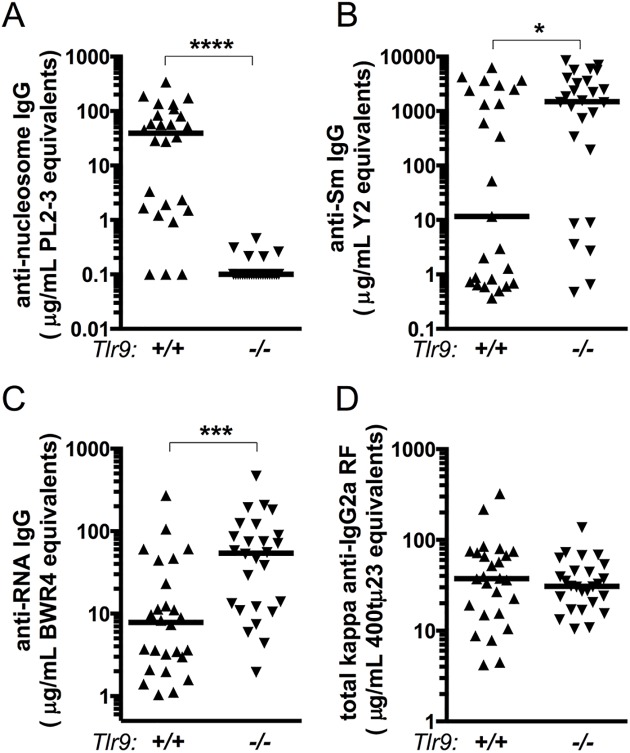
Autoantibody production in *Tlr9*^*-/-*^ MRL/+ mice. **(A)** Serum anti-nucleosome IgG autoantibodies were measured by ELISA and are expressed relative to a PL2-3 standard. **(B)** Serum anti-Sm IgG autoantibodies were measured by ELISA and are expressed relative to a Y2 standard. **(C)** Serum anti-RNA IgG autoantibodies were measured by ELISA and are expressed relative to a BWR4 standard. **(D)** Serum kappa anti-IgG2a rheumatoid factor autoantibodies were measured by ELISA and are expressed relative to a 400tμ23 standard. * p<0.05; *** p<0.001; **** p<0.0001 by two-tailed Mann-Whitney U-test.

We next evaluated MRL/+ serum by ELISA for specific autoantibodies. Consistent with the ANA results, *Tlr9*^*-/-*^ MRL/+ sera were negative for anti-nucleosome autoantibodies at 30 weeks of age, a timepoint by which 16 of 26 *Tlr9-*sufficient MRL/+ had anti-nucleosome titers greater than 10 μg/mL PL2-3 equivalents (Fig 4A). Thus, *Tlr9* is necessary for anti-nucleosome autoantibody production in both the MRL.*Fas*^*lpr*^ and MRL/+ models.

In contrast, *Tlr9*^-/-^ MRL/+ mice were more likely than *Tlr9*^*+/+*^ MRL/+ to have detectable anti-Sm titers (Fig 4B), a *Tlr7-*dependent specificity [9, 10]. 20 of 26 samples in the *Tlr9*^*-/-*^ MRL/+ group were anti-Sm-positive (titer of 50 μg/mL Y2 equivalents or greater) compared to 12 of 25 *Tlr9*^*+/+*^ MRL/+ mice (p = 0.0448 by two-tailed Fisher's exact test). Intriguingly, the median anti-Sm titer among only the Sm-positive animals was not statistically different between the two groups (p = 0.7021 by two-tailed Mann-Whitney), suggesting that *Tlr9* only affects the likelihood of becoming anti-Sm positive without affecting the actual magnitude of anti-Sm autoantibody production once tolerance to this self-antigen has been broken. *Tlr9*^*-/-*^ MRL/+ mice also had higher titers of anti-RNA than *Tlr9*^*+/+*^ MRL/+ at 30 weeks of age (Fig 4C). In contrast, and consistent with observations in MRL.*Fas*^*lpr*^ mice [10], the absence of *Tlr9* had no effect on anti-IgG2a rheumatoid factor titers (Fig 4D).

**Fig 5:**
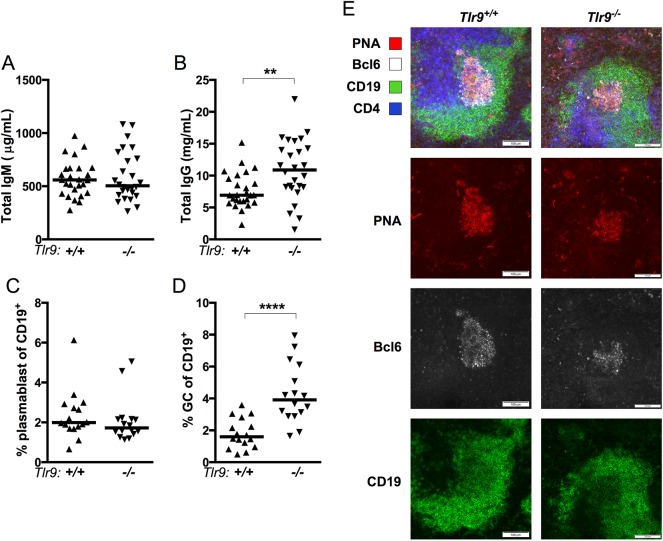
*Tlr9*^*-/-*^ MRL/+ have increased spontaneous germinal centers. **(A-B)** Total serum IgM (A) and IgG (B) were measured by ELISA. ** p<0.01 by two-tailed Mann-Whitney U-test. **(C-D)** CD19^+^TCRß^-^CD44^+^CD138^+^ plasmablasts (C) and CD19^+^TCRß^-^PNA^+^CD95^+^ germinal centers (D) were measured by FACS. **** p<0.0001 by two-tailed Mann-Whitney U-test. Data in (C) and (D) are pooled from three experimental cohorts including *Fas*^*+/+*^ mice only. **(E)** Representative sections from *Tlr9*^*+/+*^ MRL/+ (*left*) and *Tlr9*^*-/-*^ MRL/+ (*right*) mice were stained for PNA (*red*), Bcl6 (*white*), CD19 (*green*) and CD4 (*blue*). Scale bar represents 100 microns.

MRL.*Fas*^*lpr*^ mice have significant hypergammaglobulinemia compared to non-autoimmune strains, which was elevated still further in the absence of *Tlr9* [10]. We measured the titer of total IgM and IgG in serum from MRL/+. *Tlr9* did not affect the overall titer of IgM (Fig 5A). However, *Tlr9*^*-/-*^ MRL/+ mice had significantly elevated IgG titers compared to *Tlr9*^*+/+*^ MRL/+ (Fig 5B).

MRL.*Fas*^*lpr*^ and MRL/+ mice produce autoantibodies via an extrafollicular (EF) plasmablast response and also have small spontaneous germinal centers (GCs) [38, 39]. Although we were able to detect a robust EF plasmablast population in MRL/+ mice by FACS (Fig 5C) and in histological sections (S2 and S3 Figs), the proportion of CD19^+^ cells with a plasmablast phenotype did not differ with the *Tlr9* genotype on the MRL/+ background (Fig 5C).

Recent reports have suggested that *Tlr7* signals promote the GC pathway in non-autoimmune and autoimmune-prone strains [20, 40–44]. Since we observed an increase in the titer of several *Tlr7-*dependent autoantibody specificities in *Tlr9-*deficient MRL/+, we next asked whether there were differences in spontaneous GCs in MRL/+. Indeed there was a significant increase in both the proportion (Fig 5D) and absolute number (not shown) of PNA^+^ CD95^+^ GC-phenotype cells in the spleens of *Tlr9-*deficient MRL/+ compared to *Tlr9*^*+/+*^ MRL/+ mice, suggesting that intact *Tlr9* inhibits the spontaneous formation of GCs in the MRL/+ model. We were able to identify bona fide CD19^dim^ PNA^+^ Bcl6^+^ GCs in histological cross sections of spleens of both *Tlr9* genotypes (Fig 5E).

## Discussion

*Tlr9* deficiency exacerbates autoimmune disease phenotypes on several lupus-prone backgrounds, though the exact mechanisms remain unclear and are likely to involve complex interactions among multiple cell lineages. Here, by examining *Tlr9* deficiency on the MRL/+ background, we determined that the acceleration of disease observed in the absence of *Tlr9* is independent of the *Fas*^*lpr*^ mutation. This is important because, though *Tlr9* deficiency had been reported to accelerate disease in some other spontaneous lupus models, they were all driven by mutations in single alleles carried on the B6 background. Monoallelically driven models can be criticized for lack of generality as lupus in humans is polygenically driven. MRL.*Fas*^*lpr*^ mice have lupus-like disease that is polygenically driven, yet the presence of the *Fas* mutation could have in principle altered the *Tlr9*-dependency of disease.

In addition to establishing that *Tlr9* regulates lupus-like disease in a fully polygenic lupus model independently of Fas-deficiency, we revealed additional features regulated by *Tlr9* that may have been obscured by the global immune disregulation mediated by the *Fas* mutation. One striking example of this was the expansion of the myeloid compartment in the spleen of *Tlr9*^*-/-*^ MRL/+ mice, most notably the Ly6G^+^ CD11b^+^ neutrophils and to a lesser extent the CD11c^+^ dendritic cells. This contributed to a moderate *Fas*-independent splenomegaly in the absence of *Tlr9*. While splenomegaly and lymphadenopathy are major features of the MRL.*Fas*^*lpr*^ strain, they are driven predominantly by the expansion of a TCRß^+^ B220^+^ CD4^-^ CD8^-^ "DN T cell" population not observed in *Fas*-sufficient MRL/+. The myeloid expansion observed here is consistent with reports of enhanced myelopoiesis in mice engineered to transgenically overexpress *Tlr7* [45] and of monocytosis in aged male BXSB mice [46], a lupus model in which disease is driven by the *yaa* translocation that increases gene dosage of *Tlr7* among others [47]. A similar myeloid expansion has also been reported in C57BL/6 mice expressing the 564Igi IgH/IgL transgenes that encode a poorly-tolerized anti-RNA Ig [48]. Our genetic data, the serologic data here and that previously reported for MRL.*Fas*^*lpr*^ mice, as well as some *in vitro* data, indicate a stronger or qualitatively different outcome of *Tlr7* signaling in the absence of *Tlr9* in B cells [17, 49]. Since *Tlr9*^*-/-*^ MRL/+ also produce high titers of anti-RNA antibodies (as do *Tlr7*-overexpressing animals) it is unclear whether the increase in neutrophils is due to enhanced *Tlr7* signals in the absence of *Tlr9* in myeloid precursors or is an indirect consequence of autoantibody immune complex ligation of Fc receptors on myeloid cells [45, 48].

One proposed mechanism for enhanced TLR7 signaling in the absence of TLR9 is preferential endosomal delivery of TLR9 over other endosomal TLRs by Unc93b1 in cells expressing TLR7, TLR8 and/or TLR9; when TLR9 is absent, more TLR7 may enter the endosome to change the signaling threshold in response to RNA-containing antigens or immune complexes [50]. Precisely why TLR7 signaling leads to more severe disease outcomes than signaling mediated by TLR9 nonetheless remains unclear. Our data and that of others indicates that TLR7 and TLR9 control different subcategories of autoantibody, which could result in the uptake and presentation of qualitatively different antigens either by B cells directly or via FcR-mediated uptake in the myeloid compartment, as well as differentially affecting antigen clearance. Importantly, genetic deletion of both *Tlr7* and *Tlr9*, deletion of *Myd88*, or mutation of *Unc93b1* each reduce disease and autoantibody production in murine models [10, 51].

An unresolved question in autoimmune disease is the relative importance of GCs versus EF plasmablasts as the source of autoantibodies, and how TLR signaling might affect the decision between these two outcomes. *Tlr9*-dependent activation of anti-nucleosome 3H9/Vλ1 B cells on the MRL.*Fas*^*lpr*^ background proceeds via an EF route; similarly, AM14 rheumatoid factor B cells stimulated by host-derived immune complexes on the MRL.*Fas*^*lpr*^ background or by exogenously provided anti-chromatin (PL2-3) or anti-RNA (BWR4) antibodies on MRL.*Fas*^*lpr*^ or BALB/c backgrounds produce primarily Id^+^ EF plasmablasts [35, 52–54]. In contrast, AM14 B6.*Sle1*.*Sle2*.*Sle3* congenic mice have both GCs and EF Id^+^ responses in response to PL2-3 [55]. Here we find that the deletion of *Tlr9* did not abrogate the EF pathway on a repertoire-unrestricted genetic background. Although the number of cells with a plasmablast phenotype did not change in the absence of TLR9, and histologically EF plasmablasts were observed in both TLR9 genotypes, the antigen specificity of those cells was likely different, as anti-chromatin and nuclear-staining ANA were absent and anti-RNA responses were increased in the *Tlr9* deficient group. Thus, the remaining EF plasmablasts were likely generated in response to *Tlr9*-independent autoantigens, perhaps including *Tlr7-*dependent anti-RNA; we infer this due to the near-absence of EF plasmablasts in MRL.*Fas*^*lpr*^ mice lacking *Myd88* in B cells [12].

While the number of EF B cells was not changed, the number of B cells with a GC phenotype approximately doubled in the absence of *Tlr9*. Other groups have demonstrated that *Tlr9*^*-/-*^ mice on the C57BL/6 background have an increase in "spontaneous" GCs in specific pathogen free housing, which depend upon the presence of intact *Tlr7* [40, 43]. Mice that overexpress *Tlr7*, either as a transgene or in the context of the *yaa* mutation, have increased GCs on lupus-prone B6.*Sle1* or B6.*Sle1b* backgrounds [41, 42]. In two mixed chimera systems, *Tlr7*-transgene overexpressing B cells or *Tlr9*-deficient B cells each preferentially entered GCs versus WT competitors [20, 44]. While these mixed chimera experiments suggest a B cell intrinsic role of the TLRs in regulating GC formation, our previous results suggested that MyD88 signals in CD11c^+^ dendritic cells actually suppressed formation of spontaneous GCs and favored the EF response in MRL.*Fas*^*lpr*^ mice, and another group found that GCs in B6.*Sle1*.*Sle3* mice were dependent on functional plasmacytoid dendritic cells [12, 42]. Hence, the roles of TLR- and MyD88-dependent signaling are indeed complex in a variety of systems and operate differently in specific cell types, emphasizing the need for ongoing dissection of this important aspect of autoimmune disease.

In recent years we have become aware of criticism of the MRL.*Fas*^*lpr*^ model as being an artificial or poor model of lupus due to the *Fas*^*lpr*^ mutation. *Fas* mutation in humans leads to autoimmune lymphoproliferative syndrome (ALPS), which is clinically distinct from SLE but may be co-incident with SLE in some patients and often includes ANA [56]. Indeed, the B6.MRL-*Fas*^*lpr*^ (B6/*lpr*) model produces some autoantibodies but has mild renal disease [57]. Nonetheless, both MRL.*Fas*^*lpr*^ mice and the *Fas-*intact MRL/+ congenic strain meet all of the ACR criteria for lupus and have similar autoantibody profiles, though with different kinetics of disease onset. Here we demonstrated that all of the major phenotypes observed in the *Tlr9*^-/-^ MRL.*Fas*^*lpr*^ strain are recapitulated in the MRL/+ background. *Tlr9*^*-/-*^ mice progressed more rapidly to anti-Sm and anti-RNA autoantibody production, but had no anti-nucleosome autoantibodies. Naive phenotype CD4 and CD8 T cells were decreased in *Tlr9*^*-/-*^ animals and renal pathology was present at an earlier age. Indeed, for selected other genes, others and we have analyzed deficiency in both the MRL.*Fas*^*lpr*^ and MRL/+ backgrounds and in each case found similar phenotypes. These include mutations which accelerate or modify the disease course, such as *Cd274*^*-/-*^ (PD-L1^-/-^) [32], as well as those which delay or prevent disease such as B lineage or αß T lineage deletion [33, 34]. Recently we demonstrated that Fas and FasL expression on T cells, induced by cDCs, mediates the culling of extrafollicular T helper cells that support the EF plasmablast response; it is likely that the absence of this regulatory pathway at least partially contributes to the accelerated onset of autoantibody production and disease in MRL.*Fas*^*lpr*^ mice compared to *Fas*-sufficient MRL [31]. Altogether, these data validate the MRL.*Fas*^*lpr*^ model as a polygenic spontaneous lupus model that primarily differs from MRL/+ in kinetics but not quality. As more rapid kinetics makes for more efficient and economical experiments, we conclude that the MRL.*Fas*^*lpr*^ is a preferred polygenic model of lupus for certain applications.

## Supporting information

S1 Fig Enumeration of splenic lymphocyte and myeloid populations in MRL/+ mice. Cell populations were evaluated in the spleens of *Tlr9*-intact or -deficient MRL/+ mice by flow cytometry. **(A)** CD19^-^ TCRß^+^ T cells. **(B)** CD4^+^ cells expressed as a percentage of total T cells. **(C)** CD8^+^ cells expressed as a percentage of total T cells. **(D)** CD19^+^ B cells **(E)** CD19^+^ CD23/35^dim^ CD23^+^ follicular B cells. **(F)** CD19^+^ CD21/35^+^ CD23^-^ marginal zone B cells. **(G)** CD19^-^CD11c^+^I-A/I-E^+^ dendritic cells. **(H)** SiglecH^+^ CD317^+^ plasmacytoid dendritic cells. **(I)** Ly6G^+^ CD11b^+^ neutrophils.
(TIF)

S2 Fig Extrafollicular plasmablasts in *Tlr9^+/+^* MRL/+ spleen. Immunofluorescence microscopy image of representative MRL/+ spleen. Extrafollicular plasmablasts are kappa-bright (green) but IgD-negative (red) and localized within the F4/80-positive red pulp (blue). Scale bar is 50 microns.
(TIF)

S3 Fig Extrafollicular plasmablasts in *Tlr9^-/-^* MRL/+ spleen. Immunofluorescence microscopy image of representative *Tlr9*^*-/-*^ MRL/+ spleen. Extrafollicular plasmablasts are kappa-bright (green) but IgD-negative (red) and localized within the F4/80-positive red pulp (blue). Scale bar is 100 microns.
(TIF)
