# Supplementary figures and images for: Toll-like receptor 9 suppresses lupus disease in Fas-sufficient MRL Mice
Source: PLoS One. 2017 Mar 9;12(3):e0173471. doi: 10.1371/journal.pone.0173471 (PMC5344451; doi:10.1371/journal.pone.0173471)

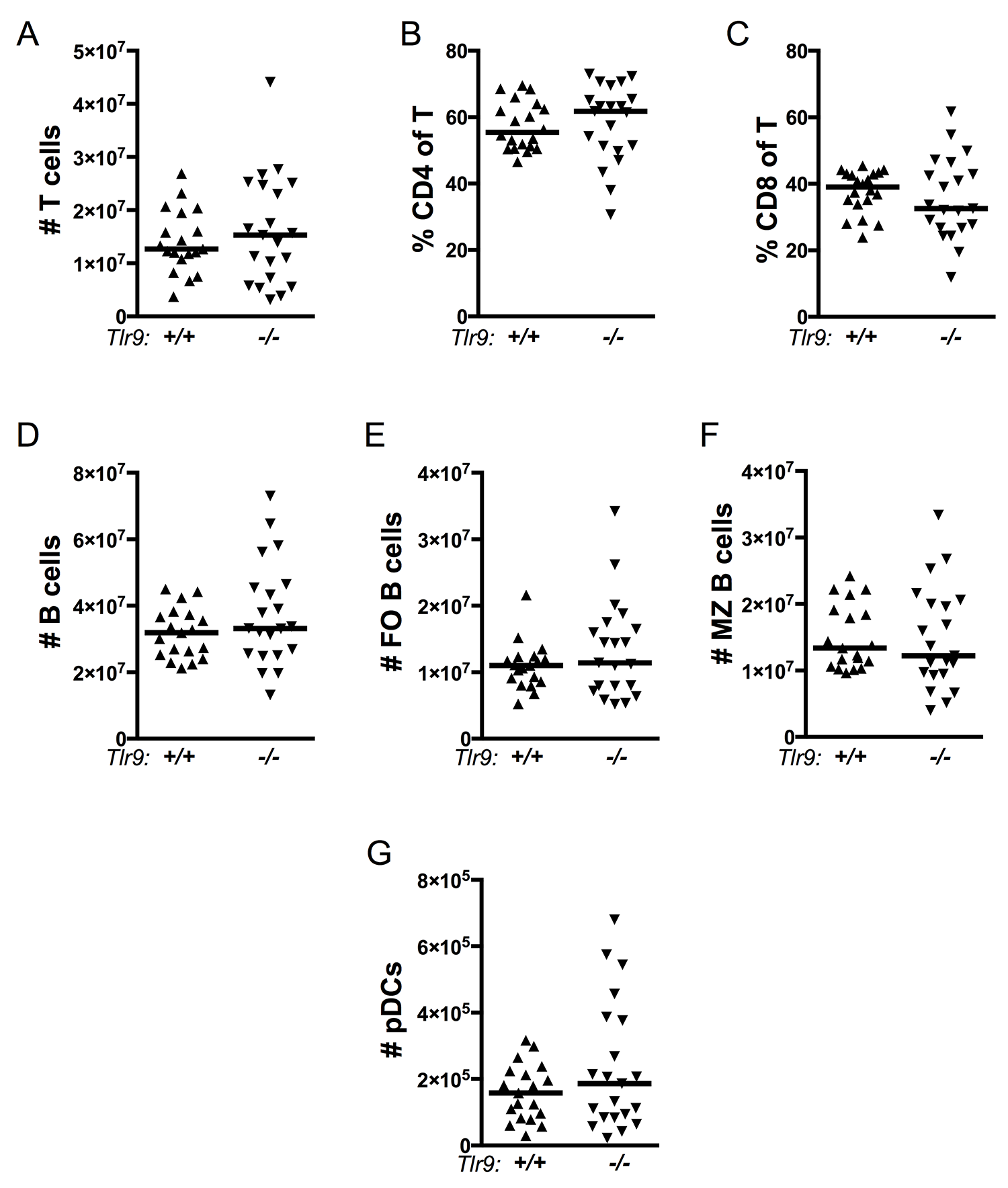

Supplement: S1 Fig — Cell populations were evaluated in the spleens of Tlr9-intact or -deficient MRL/+ mice by flow cytometry. (A) CD19- TCRß+ T cells. (B) CD4+ cells expressed as a percentage of total T cells. (C) CD8+ cells expressed as a percentage of total T cells. (D) CD19+ B cells (E) CD19+ CD23/35dim CD23+ follicular B cells. (F) CD19+ CD21/35+ CD23- marginal zone B cells. (G) CD19-CD11c+I-A/I-E+ dendritic cells. (H) SiglecH+ CD317+ plasmacytoid dendritic cells. (I) Ly6G+ CD11b+ neutrophils. (TIF) [file pone.0173471.s001.tif]

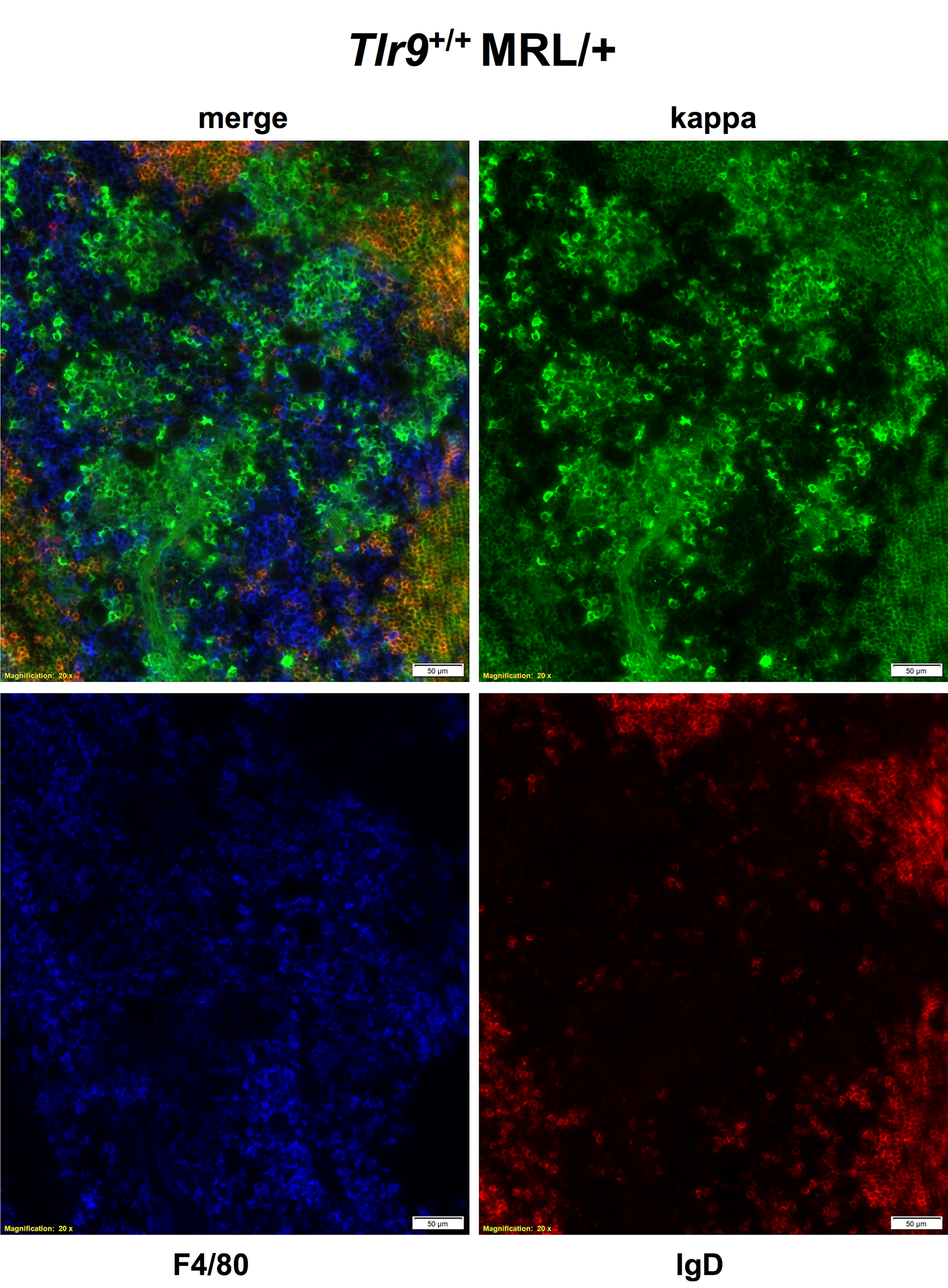

Supplement: S2 Fig — Immunofluorescence microscopy image of representative MRL/+ spleen. Extrafollicular plasmablasts are kappa-bright (green) but IgD-negative (red) and localized within the F4/80-positive red pulp (blue). Scale bar is 50 microns. (TIF) [file pone.0173471.s002.tif]

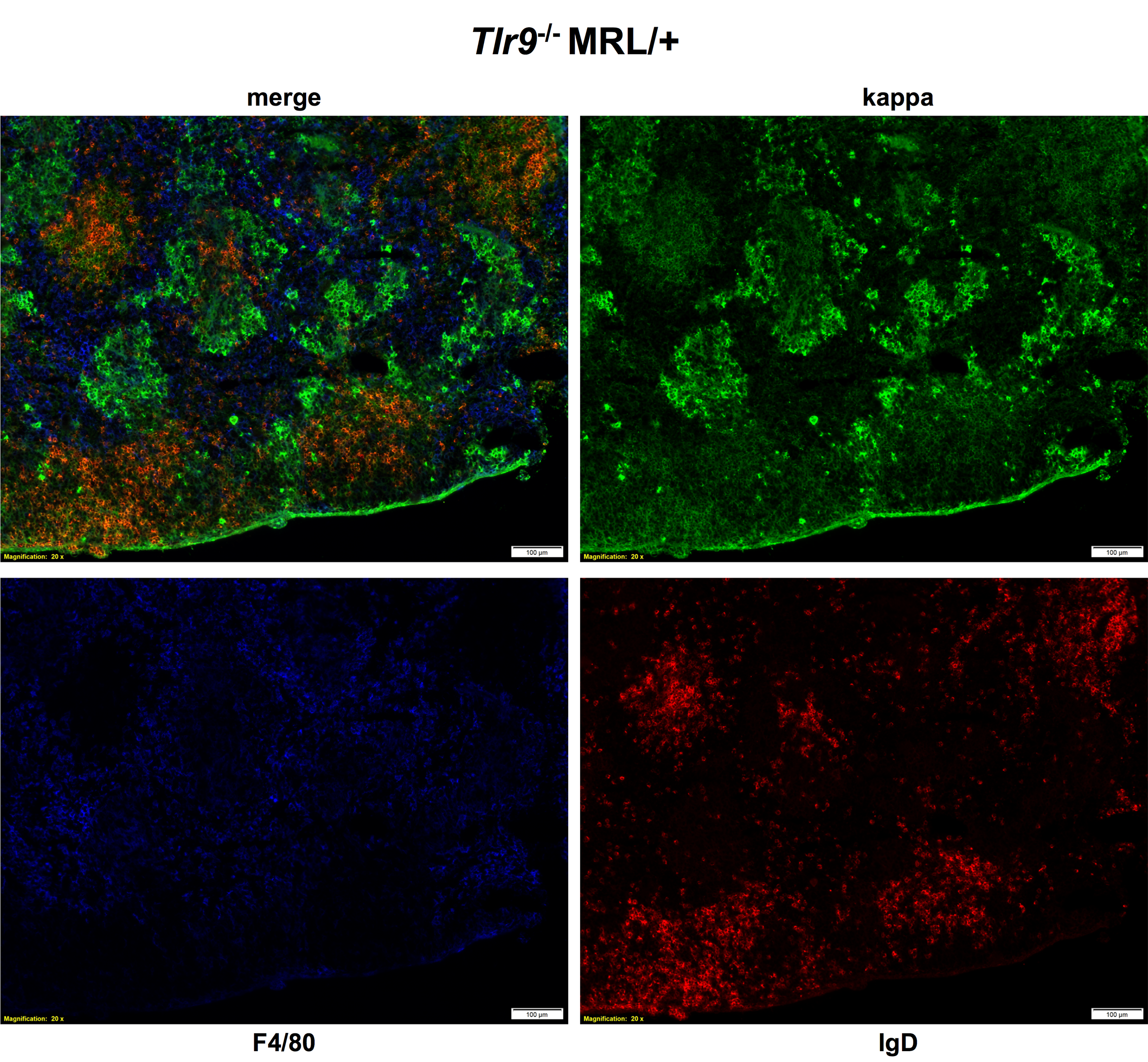

Supplement: S3 Fig — Immunofluorescence microscopy image of representative Tlr9-/- MRL/+ spleen. Extrafollicular plasmablasts are kappa-bright (green) but IgD-negative (red) and localized within the F4/80-positive red pulp (blue). Scale bar is 100 microns. (TIF) [file pone.0173471.s003.tif]
